# *‘Hu Hong’* (bad thing): parental perceptions of teenagers’ sexuality in urban Vietnam

**DOI:** 10.1186/s12889-017-4133-y

**Published:** 2017-02-28

**Authors:** Lan Anh Thi Do, Pimpawun Boonmongkon, Seung Chun Paek, Thomas E. Guadamuz

**Affiliations:** 1 Faculty of Social Work, University of Social Sciences and Humanities, Vietnam National University, 10-12 Dinh Tien Hoang street, Ben Nghe ward, District. 1, Ho Chi Minh City, Vietnam; 2 Department of Society and Health, Faculty of Social Sciences and Humanities, Mahidol University, Nakhon Pathom, Thailand; 3 Center for Health Policy Studies, Faculty of Social Sciences and Humanities, Mahidol University, Nakhon Pathom, Thailand

**Keywords:** Teenagers, Sex and sexuality, Sexuality education, Urban, Vietnam, Parental perceptions

## Abstract

**Background:**

Teenagers under 18 years old in Vietnam are considered as minors who usually lack the autonomy to make decisions. They are also sometimes viewed as contributors to social evils including crime, violence and substance use. Moreover, most Vietnamese teenagers have unsafe sex before marriage. The objective of this study is to explore the parental perceptions relating to their teenagers’ sexuality, particularly the social and cultural forces, that may hinder access to sexuality information.

**Methods:**

Guided by a Community Advisory Board (CAB), this qualitative study uses four focus group discussions (FGDs) consisting of 12 mothers and 12 fathers, as well as twelve individual in-depth interviews (IDIs) with a diverse sample of parents of teens in Ho Chi Minh City (HCMC), Vietnam. Content and discourse analysis were conducted, based on Foucauldian concepts.

**Results:**

Four themes emerged: 1) Meanings of sexuality and sexuality education, 2) Early sexual intercourse destroys teenagers’ future, 3) Teenagers are not *hu hong* (spoil/bad thing), are innocent and virgin, and 4) Policing and controlling of sexual intercourse among teens. Parents did not view their teenage children as sexual beings; those who are sexual are considered *hu hong*. Parents believed that teens need to be policed and controlled to prevent them from becoming *hu hong*, particularly girls. Controlling of sexuality information by parents was therefore common in HCMC, but differed by gender and educational levels of parents. For example, fathers more than mothers were not comfortable teaching their teenage children about sex and sexuality. Parents with higher education police their teenage children’s usage of the Internet and social media, while parents with lower education control who can be friends with their teenage children.

**Conclusions:**

Vietnamese parents in general have negative views of sex and sexuality education for their teenage children. Recognizing that many Vietnamese teenagers have unsafe sex before marriage, parents need to change their perceptions and understand the importance of comprehensive sexuality education (CSE), which are included in UNESCO, UNFPA and UNICEF-developed CSE tools.

## Background

Vietnam is a country in transition with rapid development. Youth aged 10 –19 years make up 18.7% of the almost 86 million total population in 2009 [1]. This invigoration of economic and social development began in 1986 (called the *doi moi* period) where Vietnamese culture was shaped by the influence of Western culture. Economic and social landscapes have developed profoundly, parental awareness of social changes like sexual and reproductive health may not have changed as rapidly [2].

Sexual and reproductive health issues are of concern in Vietnam. According to the Survey Assessment of Vietnamese Youth Round 2 conducted in 2009, 79% of teenage couples have unsafe first intercourse before marriage and 71% of couples did not use condoms at first intercourse. This study also showed that 44% (58% males, 30% females) of youth aged 14–25 years had premarital sex, compared with 36% of the same survey conducted in 2002. Age at first sexual intercourse has also fallen from 19.6 years in 2002 to 18.1 years in 2009 [1]. According to a 1999 report, Vietnam was 1 of 3 countries which had the highest reported abortion rates in the world, with 83 per 1,000 women aged 15–44 reported having had an abortion [3]. Ten years later in 2009, Vietnam was ranked first in Asia [4, 5]. In addition, a 2009 national report showed that abortion rates among teenagers increased 20% and among the cities in Vietnam, the highest abortion cases among teenagers were in Ho Chi Minh City, which has been increasing steadily around 2,499 cases per year [6]. Besides that, the World Health Organization reported in 2009 that 40% of new HIV infections in Vietnam were among young people [7]. Therefore, sexual and reproductive health issues are serious health concerns for teenagers in Vietnam.

These concerns have been attributed to the lack of knowledge about sexuality. Several studies have indicated that comprehensive sexuality education (CSE) is an important solution for sexual reproductive health problems in Vietnam; and Vietnamese believe that the family should be the primary source for providing it [2, 8–10]. The UNESCO-developed International Technical Guidance on Sexuality Education recommended CSE to be part of the formal school curriculum, to be delivered by well-trained teachers, and to recognized that the family is the primary source of information [11]. CSE provides opportunities for students to explore their values and attitudes, as well as develop skills in decision-making, communication, and risk reduction [12]. Furthermore, studies have shown that CSE has positive impacts and is highly effective in preventing sexual risks and promoting sexual and reproductive health among teenagers [13–17]. However, CSE programs does not currently exist in Vietnam. For example, CSE is not part of the Ministry of Education and Technology curriculum for primary, middle and high school students. Although the Ministry of Health in Vietnam has promulgated the guidelines on reproductive health services where adolescents are central, the sexuality education programs that are being implemented are not congruent to the guidelines. In the report, challenges faced by the program include traditional attitudes of parents, teachers and community members where young people should only learn SRH as it pertains to biology and reproduction [18]. Moreover, biology teachers at middle schools are uncomfortable and reluctant to discuss sexual desires and practice. The needs for accessing sexual information by teenagers are increasing following modernization [19], but teachers’ attitudes are still negative and the program has not changed to accommodate modern society [20]. Furthermore, less is known about in-depth parental perspectives on sexuality education of young people. Since previous researches have shown that CSE should involve the family, namely parents, we seek to determine parents’ feelings about sexuality education of their teenage children.

In Vietnam, parent–child communication about relationships, sexuality, and related sexual health topics are often avoided. Parents simply tell their teenage children and unmarried young adults not to have sex. Vietnamese parents often feel embarrassed talking about sensitive issues, and also hold traditional beliefs that information about sexuality, pregnancy, and contraception is not appropriate for teenagers and unmarried young adults [2, 8, 10, 21]. To date, there has not been a study of in-depth parental perceptions of sex and sexuality education in Ho Chi Minh City, Vietnam. The objective of this exploratory study was therefore to explore the parental perceptions relating to their teenagers’ sexuality, particularly the social and cultural forces that may hinder access to sexuality information.

## Methods

Ho Chi Minh City, Vietnam was chosen as the study area to represent urban Vietnam, being the largest metropolitan area with a population of 7,123,340 persons and the largest commercial center in the country [22]. A community advisory board (CAB) was created to guide this qualitative study. Informed by the CAB, focus group discussions (FGDs) and in-depth interviews (IDIs) were conducted to explore ideas, thoughts, worries and perceptions of parents on their teenager’s sexuality and social contexts that may influence teenagers’ sexuality. Participant and non-participant observations with parents of teens were conducted in Ho Chi Minh City, Vietnam between August and December 2014.

### Participants and procedures

#### Community advisory board (CAB)

Similar to our previous study [23], a 9-member CAB was created to guide the study that included (i) 5 parents (3 fathers and 2 mothers) who currently had teenage children age 15–17 years, (ii) 3 teenagers aged 15–18 years who were still in high school and (iii) a high school teacher who brought the teenagers to the CAB. The objective of the CAB was to provide feedback on the study protocol, provide advice and recommendations on the study design, IDI and FGD guidelines, and provide guidance on participant recruitment and informed consent procedures [24].

Table 1 presents demographic characteristics of research participants. Mean age of parents was 47.4 years. Parents were from various central districts of HCMC, most were employed, had not completed a bachelor’s degree, and had not participated in research studies previously. Almost all of participants’ children were currently attending public school and more than half were currently in the 12^th^ grade.

**Table 1:** Demographic characteristics of parents

	N (31)	%
Age Mean = 47.4		
Gender		
Male	13	58.1%
Female	18	41.9%
Employment		
Employed	19	61.5%
Business owner	7	22.4%
Not employed (housewife)	5	16.1%
Highest education		
Primary school	1	3.2%
Middle school	4	12.9%
High school	9	29.0%
Vocational school	2	6.4%
Bachelor’s degree or higher	15	48.5%
Experience in a research study		
Yes	4	12.9%
No	27	87.1%
Characteristics of their teenagers		
Type of School		
Private School	1	3.2%
Public School	29	93.5%
Public school (Continuing education)	1	3.2%
Grade of their teenagers		
10^th^	11	35.5%
11^th^	4	12.9%
12^th^	16	51.6%

Focus group discussions (FGDs)

Based on previous qualitative studies [23, 25–28] and suggestions from the CAB, 4 FGDs (6 participants/group) were conducted with 12 male and 12 female participants stratified by gender and level of education. Participants were recruited by snowball through key informants and members of the CAB. FGDs were conducted at the Faculty of Social Work, University of Social Sciences and Humanities, Vietnam National University - Ho Chi Minh City. Each FGD was audio recorded and lasted 2–3 h. FGDs included topics on discourses on teenager’s sexuality, sources of sexuality information (e.g., popular media, schools, health services), meanings of sex and sexuality for teenagers, and parents’ perception of their own children’s knowledge of sex and sexuality, and access to sexuality information.

#### In-depth interviews (IDIs)

A sample size of 10–15 IDIs were planned based on previous studies and data saturation [23, 25–28]. A final sample of 12 IDIs (6 females and 6 males) were conducted at the university, participants’ homes, or at public cafes; as suggested by the CAB. Of the 12 participants, 8 (4 males and 4 females) were purposively recruited from FGDs, based on their openness of sharing sensitive information and ability to speak freely on sensitive and socially “taboo” topics (e.g., masturbation). IDIs were audio recorded and lasted 45 min to almost 2 h. Interview guidelines were similar in content to the FGD to check triangulation of the data with added in-depth questions on parental perceptions on sexuality and their children’s access to sexuality information, which are topics that parents were usually shy to share during the FGDs.

#### Recruitment and data collection

The inclusion criteria for participants in this study were that participants be current parents/guardians of teenagers aged 15–17 years, reside in Ho Chi Minh City (HCMC) for at least one year and that their teenagers are current students in high schools located in central HCMC.

After consulting with the CAB, parents preferred to have FGDs on campus, either at the high school or the university, as opposed to coffee shops or restaurants because they felt less safe to talk about sensitive issues there. Furthermore, the CAB mentioned that gender and education needed to be considered because they may influence the answers of parents, suggesting that investigators separate parents by gender (male/female) and education (not/attained bachelor degree). The exclusion criteria for participants were not being able to verbally communicate in Vietnamese.

Parents in the CAB and key informants were instrumental in recruiting other parents by snowball. This method was extremely useful because parents trust other parents. In total, there were 4 FGDs (6 persons/FGD) based on gender and level of education: (1) fathers who have at least a bachelor degree and have high social position in society such as civil servants, teachers, doctors, business owners (classified as “professional father”); (2) mothers who have at least a bachelor degree and have high social position in society such as civil servants, teachers, doctors, business owners (classified as “professional mother”); (3) fathers who do not have a bachelor degree and have low social position in society such as motorbike taxi drivers, construction workers, and housekeepers (classified as “non-professional father”); and (4) mothers who do not have a bachelor degree and have low social position in society (classified as “non-professional mother”). IDI participants were chosen from FGDs if they had shared interesting ideas/issues during the FGDs, or that they have additional ideas or comments which may have been private/confidential or sensitive, and therefore they may not feel comfortable disclosing during the FGDs in front of other parents. These techniques have been used successfully in previous studies [23, 25]. Aside from this, there were parents who felt uncomfortable taking part in the FGD and so they were interviewed instead. In the end, there was a total of 12 IDIs that were conducted one-on-one either at the university or at participants’ homes. Some parents preferred that interviews be conducted in their own homes because they felt safer, more comfortable and convenient than traveling to the university. Remarkably, because the researcher visited participants’ homes, participant and non-participant observations were conducted in these homes and recorded as field notes. Here, new information was obtained because participants felt very comfortable in their own home; some giving the researcher a tour of their homes (e.g., where they slept and where their teenagers slept). Interestingly, IDI parents who scheduled interviews in their homes were all mothers and would only schedule an interview when their teenagers have left for school and when fathers were not home so to ensure privacy with the female researcher. Fathers, on the other hand, scheduled IDIs at public coffee shops near their homes because being alone in a home with a female researcher is not appropriate in Vietnamese society.

#### Procedures and materials

All parents were given a participant information sheet and an informed consent sheet that contained details about the research study including background information, research objectives, methodology, benefits and protections from harms. Moreover, parents were also given a short demographics questionnaire before the FGDs in order to assess characteristics of parents and their teenagers. FGD and IDI field guides were reviewed and approved by the CAB and subsequently used in all FGDs and IDIs. Each parent who consented to participate was compensated with 200,000 VND (~US $10) for his or her time and travel. All FGDs and IDIs were voluntary and anonymous and parents may stop or cancel the FGD/IDI at any time.

The research protocol was reviewed and approved by Mahidol University Institutional Review Board.

#### Data analysis

Data collection and analysis in qualitative research is an ongoing process once the field work begins and continues until it ends. First, data came from several sources including: (1) field notes from CABs, FGDs, IDIs, informal interviews and the first author’s daily ethnographic diary; and (2) voice recorders that was transcribed immediately after each FGD/IDI. Then, all text from field notes and transcripts were translated verbatim from Vietnamese to English by the first author and re-checked by a Vietnamese teacher who is fluent in English to ensure translation accuracy. Second, the authors read at least twice each transcript. Third, each author independently coded each transcript and field note. Fourth, codes were then compared and emergent themes were discussed among the authors for congruence [29]. Any differences among authors were resolved through discussion meetings. Theoretical approaches (Foucauldian concepts of discourse and sexual analysis) were used to interpret and synthesize major themes from our conceptual framework. All identifying information from participants were removed during analyses and not presented in this article. The above procedures were meant to ensure data and methodological triangulation, as well as to minimize bias and improve the validity of findings [30].

## Results

### Meaning of sexuality and sexuality education

#### Sexuality is sexual intercourse

Most parents did not understand or were aware of CSE; they thought of sexuality as only sexual intercourse. For example, the Vietnamese word for sexuality is *tinh duc* which most participants associate with sexual intercourse, which is considered only appropriate after marriage. Further, parents said that sexuality is ‘normal’ for adults who are married as it is necessary for reproduction. These understandings were generally similar across fathers and mothers, and different levels of education.

“*Sexuality is sexual intercourse in Vietnam context”* Mother professional FGD

*“For adults who got married like us, it must be sexuality in marriage. Without sexuality, marriage will be broken”* Father non-professional FGD

*“Sexuality is normal for spouses like working, people need to have [it]… life needs to have sexuality”* Father non-professional FGD

*“…sexuality is for survival of the species…it is a life’s need and yin-yang harmony”* Mother non-professional FGD

*“Sexuality is a human’s instinct. It comes from human needs and lead to sexual intercourse because of expression of feelings and action”* Mother non-professional FGD

#### Sexuality is for adults, not for teenagers

Parents said that sexuality did not exist among teenagers because they were still in schools and their primary duty was to study. If they had sex, their future would be destroyed. However, some parents thought that they should talk to their kids about sex/sexuality because it is impotant. In reality, however, parents felt uncomfortable talking about sex/sexuality with their children. Moreover, their teenagers did not ask or discussed with them about topics related to sexuality. Parents also assumed that their teenagers had already learned about sex from school. And so parents would only talk about it if their teenagers asked or brought up the issue for discussion. One father in non-professional FGD stated that:

*“I think I should teach my kids about it [sex] but my kids have not ever asked me, so I have not talked to them yet”*

Another reason parents did not want to talk to their teenagers about sexuality was because they only viewed sexuality as negative for teenagers, that sexuality would have bad consequences for their teenagers. One non-professional father FGD said:

*“Sexuality is normal for us [adults], but not for my kids who are not adults,… if I talk about that to my kids too much means that ‘I am showing a way for deer [innocent animal] to run’”*

Another non-professional father IDI had a similar opinion:

*“Sexuality in teenagers has many bad consequences… It is not good for my son’s future… I told my son that ‘now you are still sitting on the chair of school, you should not do it, you should keep far away from it [sex]”*

#### Sexuality education is the responsibility of the school and the mother

Most parents thought that it was quite difficult to talk about sexuality with their teens and so sexuality should be taught at school. A non-professional father IDI stated:

*“Nowadays, in the school, they have already taught them. The school should teach our kids about that [sexuality]”*

At the family level, however, sexuality education is the mother’s role. Parents indicated that mothers are easier to talk to teenagers than fathers about sexuality and other sensitive topics. One professional father in the FGD stated:

*“Mom is close and [the] major [person] in talking to kids [about sexuality] than Dad”*

Another non-professional father IDI added: *“For me, I think Mom is very helpful for kids but my wife and me divorced so I try to get information to talk to my daughter but she did not ask me [bring it up]....”*

A non-professional mother from an FGD confirmed that *“the fathers are rare; they are always hesitating, reluctant and shy to talk about this subject [sexuality] for teenagers… mostly mothers are closer to kids than fathers”*

### Early sexual intercourse detroys teenagers’ future

Parents felt that when teenagers involved themselves in sex and sexuality too early (before marriage or at least before graduation from university), they would have difficulties, would drop out of school, and would ultimately be unhappy.

*“…early sexual intercourse without preparation will bring much difficulties for teenagers in the future such as quiting school, not be a good person, unhappiness in life and even having babies that will also be bad like them”* Father non-professional FGD

*“Teenagers with early sexual intercourse will not be successful, will be a burden, and a tragedy for society, especially teenage mothers who cannot afford to take care of their babies....”* Father non-professional FGD

Almost all parents thought that teenagers had already accessed enough information by themselves via the Internet, other social media, and from school. Parents said that it was quite difficult to talk to their teenagers about sexuality, that information should come from school, should be a major subject in school and should be provided from experts of sexual and reproductive health. Regardless, they tried to give advice to their kids like examples from movies about unsafe pregnancy or examples for “bad” girls who had not finished school because of unplanned pregnancy or who preferred to go out rather than study. For girls, parents reinforced the ideas of virginity, hygiene, purity, and love. For boys, they mentioned focusing on studying rather than playing games, not making friends with “bad” boys and not being seduced by “bad” girls or gay men.

### Teenagers are not *hu hong*; instead are innocent and virgin

Parents perceive their teenagers as pure, innocent and not interested in sex and consequently, sexuality information. The ways parents talked about their teenagers are as if they are innocent good children whose only job is to study and go to school. Parents are at ease when they know that their teenagers do not have girlfriends or boyfriends. One mother in the professional group stated:

*“My son prefers playing games only, he does not like to watch romantic movies or going out with girls. He does not have a girlfriend. He looks like a kid. He does not know everything about that [sexuality]....”*

Most parents did not accept that their teenagers were having sex. From parental perspective, sexuality is immoral and not good for them. Having sex will disrupt teenagers’ schooling and subsequently, their future. It will lead to unexpected burdens in their lives.

On the other hand, parents accepted sexual intercourse within marriage and only among adults. Adult in this context did not merely mean age 18 years and over as recognized by Vietnamese law, but rather it meant having completed high school or university, currently working and being able to support themselves. One mother in the non-professional group pointed that *“I will let him do whatever he wants with the condition that he graduates from high school and passes the national examination to enter University.”*

Another reason that makes parents feel strongly that teenagers are not sexual beings is that they worry their teenagers will break the law and end up in prison if they have sex with other teenagers under 16 years. This concern follows the current Vietnamese law where anyone having sex with children under 16 years will go to prison. One non-professional mother FGD said that *“there is a case, who was a son of my friend, he [17 years old] had to be in prison for 10 years because of having sex with a teenage girl under 16 years [13 years old]…”.*

This reason also made professional mothers worry about their sons. A professional mother IDI said: *“I do not forbid my son to have girlfriends within pure love, if they have sexual intercourse then something happened, my son could be in prison. I am so worried about that.”*

A definition of *hu hong* was pointed out many times during FGDs and IDIs. *Hu hong* which roughly translates to “spoil” or “bad thing” is a term used for bad teenagers who are sexual beings; for example, those who take care of their appearances, altering their bodies and displaying their sexuality in public. This term was used quite frequently to emphasize bad girls. This heavy emphasis for girls pointed out how this term is negatively used for girls, but may not be as negative for boys. A non-professional father IDI stated:

*“The girls who prefer to show their bodies, dye their hair, wear fit-pants are no good at studying, they just good at playing outside with boys… That kind of girl is called hu hong…For teenage girl, this is very serious because it is negative…”*

For boys, *hu hong* can mean naughty boys who are strong, muscular, smoking, playing sports (those that prefer playing than studying). One father in non-professional FGD said *“For boys, it is acceptable that they prefer to play games than study. It is normal for boys to be “hu hong” which means naughty, manly and strong…”*Another mother added more in the FGD that *“For teenage boys who are too weak and gentle, we will blame him as ‘be de’ [gay/girly/less masculine boys]… teenage boys should be strong so that they will not be attacked/persuaded by gay men…”*

These findings point to the social differences in the meaning of *hu hong* by gender. While *hu hong* is generally a negative term to describe teenagers, it is meant to be much more negative for girls than for boys. Girls are “bad” if they display their bodies, go out with boys, and if they do not study. These beliefs highlighted how girl’s sexuality is still policed and controlled in Vietnamese society and the perpetual dominant belief that boys will be boys, but girls need to be innocent and pure in order to be “good” girls.

### Policing and controlling: preventing sexual intercourse among teens

*“When my son was in grade 8, his peers gave him a VCD [video compact disk] of sex movies and then he watched it, … one day his teacher found out and my son became scared that his teacher would talk to me about it so he wrote a letter to his teacher saying that if I knew [about it], I would kill him, and he would die”*

*Father non-professional FGD*

The above story is an example of fear by a teenage boy toward his father. He knew that his father would not approve of him accessing this VCD that contained sexual activities. There were many ways parents controlled and policed sexual information so that their teenage children would have limited access to this information. Parents assumed that teenagers would not be bad if they paid attention and policed them as much as they can. Parents also fear the rapid development of mass media which may provide easier access for their teenagers, thus challenging their authority and control of sexual information.

One example of control is to not let their teens go out to parties, thus limiting their opportunities to do “something wrong.” One professional mother IDI said: *“I will not allow my son to join the birthday party at his friend’ house, I try to make him forget that event by requesting him to do something on that day.*

In another FGD, parents even worried when their teenagers requested to go to school by themselves or to go to their friends’ house after school. One professional father in the FGD said *“When my daughter was in grade 10* ^*th*^ *, she requested to go to school by herself with a bicycle. I was so worried, and so I followed her from behind until she reached her class…”*

#### Masturbation

*“My wife does not want my son [15 years old] to sleep at another place. We all still sleep in the same bedroom because she wants to supervise and know what he does before he goes to bed. We know that he always does masturbation before sleeping… but when she supervises, he cannot sleep, he has to wait for us sleep and then he will do it until he can sleep. I told my wife to leave him alone and let him masturbate, but she does not listen to me, she wants to supervise him as much as she can….”*

*Father non-professional IDI*

In this example, the mother took comfort in sharing the same sleeping space as her son so that she could observe, control and police her son. Although the father understood his son’s desires, the mother did not. Remarkably, these parents mentioned masturbation in the context of their son only, not their daughter, even though they were similar in age and lived in the same house. In general, parents felt that by policing and controlling their teenagers’ sexuality, they were being responsible parents. Furthermore, to promote abstinence for their children, some parents even practiced abstinence as an example for their children. One non-professional mother IDI explained:

*“Because of economic condition, our family had to sleep in the same room, we [parents] have to control our sexual desires to avoid influence on our teenagers. We had to wait until our kids have gone to bed, and sometimes we just don’t have sex to ensure that our kids cannot see it. Since our kids grow up [became teenagers], my husband and I have to limit our own sexual intercourse.*

#### The Internet

Most parents believed that policing and control were best for their teenagers who were still in schools. Moreover, parents policed and controlled their kids by forbidding them to use mobile phones late at night, and telling their teenagers not to make friends with ‘bad’ teenagers (e.g., those who prefer going out than studying, who are lazy and who take or are addicted to drugs). However, there are distinct kinds of policing and control for their kids between non-professional parents and professional parents. For example, highly educated mothers and fathers blocked websites they deem to be of a sexual nature or where teenagers are known to meet and chat, and regularly check their kids’ Facebook and Gmail accounts, including text messages on smartphones. Several parents knew their kids’ passwords from either reading their kids’ diaries or by observing their kids typing in their passwords. Parents also regularly checked personal activities and personal belongings of their teenagers to confirm that their teenagers are not doing anything wrong and are “on track.” A professional father IDI shared: *When she went to school, I turn on the computer and check her [web browser] history and what information she had accessed…”*

A professional mother FGD said: *“I hired an IT person to block ‘black’ websites [websites known to have pornographic materials] to prevent my son and daughter from accessing them…”*

Another professional mother IDI added: “*He was so naive, I opened his Facebook account and checked his email and then confronted him about his girlfriend…”*

While similar, non-professional mother and father had a different strategy of policing and control. Some parents placed the computer in the shared living room to easily observe their daughters/sons’ Internet usage, or kept the computer in a locked room and its use under direct supervision.

One non-professional mother IDI said that *“….I placed the TV and computer in the living room, if he [teenage son] wants to use, he has to come to the living room so I can supervise him… for Internet I allow him to access 1–2 h/week under my observation…”*

One non-professional father IDI had another way, he said: *“… My children had to add me/friend me on Facebook. If they do not, I will cut the Internet at home… Thus, I can follow [their] activities on Facebook…”*

For professional parents, their policing and control is more indirect and deceptive (e.g., sneaking into Facebook, checking email accounts and web browser history), whereas for non-professional parents, the policing and control is more direct and less deceptive (e.g. placing the computer in a locked room, asking their kids to be friends on Facebook).

## Discussion

The most prominent findings of this exploratory study were that parents rarely talk about sex with their teenage children. Moreover, they do not like to talk about sex in general, even with the researcher. During data collection, the researcher recognized that parents felt uncomfortable discussing sexuality issues [8], even refusing to attend focus group discussions once they learned about the study’s objectives. This study confirms findings of previous research conducted in Hanoi (North Vietnam) and Nha Trang (South Central Coast of Vietnam) where parents did not talk with their children about sensitive topics like sexuality [2]. In addition, this study confirms that parents’ perceptions have not changed, even when Vietnam is changing socially and culturally in the current rapid economic development [31].

The rapid changes of socio-economic contexts in the biggest city in Vietnam, where the study was conducted, may have influenced the perceptions of parents on their teenagers’ sexuality. According to Foucault, knowledge and truth are different among and between institutions, defined “as a relatively enduring and stable set of relationships between different people, and between people and objects” including public institutions and private institutions [32]. Applying this concept to the Vietnamese context, institutions like schools and the family are important gatekeepers that may police and control access to knowledge for teenagers regarding sex and sexuality. But, at the same time, under today’s rapid development in technology and relatively easy access to technology by teenagers (e.g., affordability of mobile phones), the “power” of institutions to police and control access to knowledge may become more and more obsolete as teenagers find other ways to access knowledge (e.g., Internet).

Our findings suggest that Vietnamese parents have negative views of teenagers’ sexuality and tend to define sexuality as sexual intercourse. In Vietnam, sexuality is considered sensitive and taboo [20]. Parents believe that discussing sexuality will lead teenagers to sexual experimentation and sexual practice [33]. Parents therefore chooses to ignore their teenagers’ needs and rights to be sexual beings [10]. Teenagers, on the other hand, want to access sexuality information as soon as possible. According to International Planned Parenthood Federation (IPPF), teenagers have needs and rights to access comprehensive sexuality education not only in high school, but beginning in primary school [34]. In Vietnamese contexts, teenagers have their own needs to access sexuality information but they do not have rights to do it. One non-professional father said that rights have to be based on cultural contexts and parental permissions are always needed. Also, parents feel that they are always right and they have the power to enforce their own beliefs. This logic is similar to what Foucault said, that parents often create their own meanings of the truth, when it is convenient for them and when that truth is different from their kids, since they have the power to do it [35].

Our study supports earlier findings that sexuality is considered taboo in the Vietnamese context. In the past, sexuality information is forbidden by government, schools and family [2]. In addition, there has always been a gap between parents and children in communication, especially on sensitive topics like sexuality or sexual and reproductive health [10, 36, 37]. Therefore, parents in this study rationalize that by controlling and policing, they are taking on the responsibility of their teenagers. Parents do this in different ways, including limiting access to certain information on the Internet, limiting time using the Internet and television, and even controlling who becomes friends with their children.

In the end, parents do not want their teenagers as sexual beings, instead they want their teenagers to follow their version of the “truth” and to teach their children that sexuality is sinful and taboo, and something that should be avoided [35]. In parallel, parents cannot understand their teenagers’ sexual subjectivity and would rather pretend that their teenagers are “good” and obedient, even when, in reality, teenagers are constantly negotiating their parents’ version of the truth and will follow only a part of what their parents tell them [38]. Moreover, parents thought that the school should be tasked with providing sexuality education, not them. Parents believed that the schools will provide their children the “right” information about sex, which of course means sex for reproduction only. This implies that when sex is for pleasure, for example, it is “wrong” or considered a “bad” activity. However, parents view that their role is to police and control sex and sexuality information as much as they can when their teenage children are outside the school. Indeed, teenagers said that parents should be more active in talking to them about sex and sexuality. Interestingly, younger parents (under 40 years old) in our study were more acceptable of their children accessing sexuality information than older parents, who tended to have more traditional views.

While parents believed that schools can provide the “right” sexuality information for their teenage children, these schools are also traditional institutions and are limited in providing sex and sexuality education. For example, one high school teacher complained that she and other teachers cannot even properly analyze the sexual characteristics/meanings in the poem/literature because the high school administration did not allow them. Another example is when a mother in our study, who happens to be a medical doctor, was invited to teach about sex education in high schools. The principal told her that she should not teach about sexuality too much, just focus on reproduction in general and was given only 30 min to talk to the entire student body at the high school. If we consider Foucault’s concept of knowledge and power within the context of the current transitional economy in Vietnam, which tends to value hard-working laborers and individualism, we will observe that there is a false sense of power among parents and school administrators. The power is in fact being shifted to teenage children who negotiate the “truth” given to them by teachers and parents and then explore and navigate their own truth about sex and sexuality through various sources currently accessible to them (e.g., Internet, porn, etc.) [32].

### Research implications

This research informs future interventions where parents need to be trained for the benefits of comprehensive sexuality education in order to change their perceptions of teenagers’ sexuality. In addition, parents and teachers need to understand that they can no longer police and control sexuality knowledge and information on teenagers in the current age of technology since their teenagers will find alternative sources of information. Finally, more research needs to examine the relationship between limited and incorrect sex and sexuality information among teenagers and the rise in abortion rates, unwanted pregnancy and unprotected sex in Vietnam.

### Recommendations

#### Comprehensive sexuality education in schools for parents, teachers and teenagers

According to the several United Nations agencies, CSE should be provided to promote sexual and reproductive health for young people in schools from kindergarten to upper-secondary or high school and CSE should either be a major subject in the curriculum or be included as part of every class (e.g., chemistry, history, foreign languages, etc.). In addition, since schools are places where teenagers spend most of their time, teachers are important key partners if CSE is to be successful [12, 39]. Studies have shown that CSE in schools do increase knowledge and awareness for teenagers, which in turn reduces their sexual and reproductive risks [13–17]. While parents in this study exercised their power to control sexuality information for their teenage children, they still realized the importance of sexuality education and reproductive health for their teenagers. Parents want their children to learn from schools and stressed the importance of having an expert to talk to their children about sexual and reproductive health.

At the family level, parents play an important role in shaping key aspects of their children’s sexual identity, and sexual and social relationships. In general, effective CSE programs recognize parents and the family as importance sources of sexuality information, support and care. Therefore, including parents and families in sexuality education is vital in the International Technical Guidance on Sexuality Education [12]. Parents pointed out that they need to know how to better communicate with their teenagers about sex and sexuality and how to teach their teenagers about sexual and reproductive health at home.

#### A safe and private space for CSE in schools for students, teachers and parents

Some professional parents in our study did not agree with the way the schools currently provide sexuality information for their teenagers. For example, allocating only 30 min to provide sexuality information to all school students at once is not an effective way. Parents hoped that their teenagers can access information as much as they can at schools where a safe and private space is necessary. Some parents suggested that schools should have a counseling room for teenagers who want to talk about problems relating to studying, love, family and even unwanted pregnancy—basically anything teenagers cannot share with their parents. This room can also be used to teach CSE for parents and teenagers, as well as train teachers on CSE.

#### A reliable campaign to provide correct information about sexuality via the Internet

Parents recognized the importance of the Internet. And so, they want reliable and easily accessible sexuality information on the Internet for themselves and for their children. One professional father said:

*“I hope that schools, organizations or you – researchers—can provide a website or blog or Facebook page to provide correct information for teenagers to access [sexuality information] because the information on the Internet is so complicated and parents cannot manage it”*

#### Limitations

This exploratory study has some limitations. The sample size in the study was small, which may have affected the generalizability of findings. However, the objective of this study was to contextualize the perceptions of sexuality education among parents of teenage children in order to guide a larger quantitative study. And so while the sample may have been small, there were still variability among study participants, including participants with diverse educational backgrounds, gender and residents of several districts in central Ho Chi Minh City, as opposed to previous studies in Vietnam where participants had less demographic variability [8, 10, 21]. Furthermore, this study was guided by a community advisory board to ensure appropriateness to the contexts of local communities, including appropriate recruitment strategies and adaptation of the interview guide. Another limitation is that the study did not include teenagers themselves, but only their parents. While this may be a limitation for comparison analyses, the objective of this study was to learn about parental perceptions and so including only parents was appropriate for this exploratory study.

## Conclusion

Urban Vietnamese parents try to police and control their teenagers closely, through various strategies—depending on whether parents are professionals or non-professionals. In general, parents have negative beliefs about sexuality: that sexuality means sexual intercourse only, teenagers are not sexual beings and sexuality education will lead to sexual intercourse among teenagers. There is a need for comprehensive sexuality education for parents, teachers and teenagers. A campaign should be created to provide corrected information about sexuality on the Internet since this is a place where teens go for information, hang out and meet new friends. Government should consider comprehensive sexuality education in its current school curricula, as well as provides opportunities for parents of students to be involved. Moreover, parents need to realize that their teenagers having exposure to CSE does not mean that they will become *hu hong* (bad/spoil things), or that they will engage in *te nan xa hoi* (social evils/social vices). However, the government also needs to play its part and not encourage or reproduce this kind of belief/social norm. For example, there still exist a Bureau of Social Evils Prevention and Combat within the Ministry of Labour, Invalids and Social Affairs that deals with sex workers, drug users, human traffickers and people living with HIV/AIDS. Having a bureau of social evil reproduces social stigma for these groups. While long overdue, changing the name of the bureau would be a welcomed structural change from the Vietnamese government.

## Abbreviations

CAB: Community advisory board
CSE: Comprehensive sexuality education
FGD: Focus group discussion
IDI: In-depth interview
